# In depth behavioral phenotyping unravels complex motor disturbances in *Cstb^−/−^* mouse, a model for progressive myoclonus epilepsy type 1

**DOI:** 10.3389/fnbeh.2023.1325051

**Published:** 2023-12-21

**Authors:** Eveliina Pollari, Saara Tegelberg, Harry Björklund, Reetta Kälviäinen, Anna-Elina Lehesjoki, Antti Haapalinna

**Affiliations:** ^1^R&D, Orion Pharma Corporation, Turku, Finland; ^2^Folkhälsan Research Center and Medicum, Medical Faculty, University of Helsinki, Helsinki, Finland; ^3^Epilepsy Center, Neuro Center, Kuopio University Hospital, Kuopio, Finland; ^4^Institute of Clinical Medicine, University of Eastern Finland, Kuopio, Finland

**Keywords:** EPM1, cystatin B, myoclonus, behavioral phenotype, startle response, hyperactivity, elevated plus maze, CatWalk

## Abstract

Progressive myoclonus epilepsy type 1 (EPM1) is an autosomal recessively inherited childhood–adolescence onset neurodegenerative disease caused by mutations in the cystatin B (*CSTB* gene). The key clinical manifestation in EPM1 is progressive, stimulus-sensitive, in particular action-induced myoclonus. The cystatin B-deficient mouse model, *Cstb^−/−^*, has been described to present with myoclonic seizures and progressive ataxia. Here we describe results from in-depth behavioral phenotyping of the *Cstb^−/−^* mouse model in pure isogenic 129S2/SvHsd background covering ages from 1.5 to 6 months. We developed a method for software-assisted detection of myoclonus from video recordings of the *Cstb^−/−^* mice. Additionally, we observed that the mice were hyperactive and showed reduced startle response, problems in motor coordination and lack of inhibition. We were, however, not able to demonstrate an ataxic phenotype in them. This detailed behavioral phenotyping of the *Cstb^−/−^* mice reveals new aspects of this mouse model. The nature of the motor problems in the *Cstb^−/−^* mice seems to be more complex and more resembling the human phenotype than initially described.

## 1 Introduction

The cystatin B-deficient (*Cstb^−/−^*) mouse, generated by targeted disruption of the *Cstb* gene resulting in complete loss of CSTB protein, was described more than 25 years ago as a model for progressive myoclonus epilepsy type 1 (EPM1, Unverricht-Lundborg disease, OMIM #254800; Pennacchio et al., 1998). EPM1 is a neurodegenerative disease caused by bi-allelic partial loss-of-function mutations in the cystatin B (*CSTB*) gene (Pennacchio et al., 1996; Joensuu et al., 2008), a potent and reversible inhibitor of cysteine cathepsins (Turk and Bode, 1991). EPM1 is characterized by progressive, stimulus-sensitive myoclonus and tonic–clonic epileptic seizures with onset typically between 6 and 15 years of age. While the seizures are usually well controlled by antiseizure medication, the myoclonus is treatment resistant and severely incapacitating (Lehesjoki and Kälviäinen, 2020) and increasing negative myoclonus leads to falls and inability to walk. Some years after the onset, the patients develop ataxia, incoordination, intentional tremor, and dysarthria, but their cognition remains mostly within normal range. Total loss of CSTB protein in humans results in neonatal onset developmental encephalopathy with progressive microcephaly, and hypomyelination (Mancini et al., 2016; O’Brien et al., 2017).

The behavioral phenotype of the *Cstb^−/−^* mouse was reported to recapitulate the key features of EPM1 disease: myoclonic seizures, and ataxia, with widespread apoptotic death of cerebellar granule cells being characteristic for the mice (Pennacchio et al., 1998). After this initial description, no systematic or more detailed behavioral phenotyping in *Cstb^−/−^* mouse have been published, but the focus of research has been on studies aiming at understanding the mechanisms associated with deficient CSTB function on tissue, cellular and molecular level. These studies have unraveled that the progressive degeneration in *Cstb^−/−^* mouse brain is not limited to cerebellum but is widespread, involving both gray and white matter in a pattern seen in EPM1 patients (Tegelberg et al., 2012; Manninen et al., 2013, 2014). The earliest reported signs of CSTB-deficiency in mice involve defects in neurogenesis and interneuron migration during embryonal development (Di Matteo et al., 2020; Daura et al., 2021), with altered synapse physiology, GABAergic signaling, and prevalent loss of GABA interneurons reported in post-natal brain (Joensuu et al., 2014; Penna et al., 2019; Gorski et al., 2020, 2023, Buzzi et al., 2012). A key manifestation in *Cstb^−/−^* mouse brain is glial activation followed by marked neuroinflammation (Lieuallen et al., 2001; Tegelberg et al., 2012; Okuneva et al., 2015, 2016). Microglial activation in *Cstb^−/−^* mouse brain appears before clinical onset at around 2 weeks of age (Tegelberg et al., 2012). Microglia show compromised functional properties *in vitro* (Okuneva et al., 2015) and impaired phagocytosis of apoptotic cells in the brain (Sierra-Torre et al., 2020). Microglial activation is followed by progressive astroglial activation coinciding with a clinical onset at around 1 month of age (Tegelberg et al., 2012). One emerging pathogenetic mechanism in *Cstb^−/−^* mouse brain is compromised energy metabolism reflected by impaired mitochondrial respiration in developing neurons (Daura et al., 2021) and in cerebellar synaptosomes (Gorski et al., 2023).

The behavioral phenotype of the *Cstb^−/−^* mouse is strongly dependent on the genetic background with mice in mixed background having a milder phenotype (Pennacchio et al., 1998). In isogenic 129Sv background, *Cstb^−/−^* mice develop myoclonic seizures by 1 month of age (Pennacchio et al., 1998). In mixed background myoclonus are observed considerably later, at 8–9 months (Pennacchio et al., 1998; Houseweart et al., 2003). The myoclonic events occur predominantly during sleep and begin with twitching of whiskers, ears, and tail, before proceeding to shaking of the torso and limbs, and muscle jerks powerful enough to catapult the mouse in the air (Pennacchio et al., 1998; Houseweart et al., 2003). The initial phase of these episodes has been associated with abnormal cortical activity in electrocorticographic recording, while the later generalized myoclonic movements showed no specific discharge pattern (Pennacchio et al., 1998). Although *Cstb^−/−^* mice show no spontaneous convulsive seizures, or photosensitivity (Pennacchio et al., 1998), which are characteristic to EPM1 patients (Lehesjoki and Kälviäinen, 2020), they exhibit latent hyperexcitability and are susceptible to kainite induced epileptic-like seizures (Franceschetti et al., 2007).

Results from other behavioral manifestations in *Cstb^−/−^* mice have only been reported in animals with mixed background. The mice show disturbed motor coordination with poor performance on both still and rotating (2 rpm) rotarod from 4 months of age (Pennacchio et al., 1998; Kaur et al., 2010). At 6 months of age, the mice show wide-based gait and balance deficits when challenged to walk on uneven surfaces (Pennacchio et al., 1998; Houseweart et al., 2003). These findings have been interpreted as signs of ataxia, mirroring the symptoms described in EPM1 patients (Lehesjoki and Kälviäinen, 2020), although ataxia in EPM1 patients is mostly caused by myoclonus.

As reports on systematic behavioral phenotyping of the *Cstb^−/−^* mouse model are limited to only one study published more than 25 years ago (Pennacchio et al., 1998), more detailed phenotyping with new technology would allow for better quantitation of the symptoms and could provide new information on their occurrence in parallel to the neuropathological processes described in detail in the mouse. We here describe results from in-depth behavioral phenotyping of the *Cstb^−/−^* mouse in pure isogenic 129S2/SvHsd background covering ages from 1.5 to 6 months. In line with the previous report, the mice had progressive myoclonus. While previous reports of myoclonus in *Cstb^−/−^* mice have been descriptive we describe here a novel software-assisted quantitative method to analyze the occurrence of myoclonus in more detail. Our behavioral assays revealed that the mice were hyperactive, agitated and impulsive, and showed reduced startle response, problems in motor coordination and lack of inhibition. We were, however, not able to demonstrate an ataxic phenotype in them.

## 2 Materials and methods

### 2.1 Mice and experimental design

The CSTB-deficient mouse strain used in this study is 129S2/SvHsd-*Cstb^tm1Rm^*, derived from the Jackson Laboratory strain 129-*Cstb^tm1Rm/J^* (stock no. 003486; https://www.jax.org/strain/003486; Pennacchio et al., 1998). The genetic background of the mouse colony was refreshed annually with new 129S2/SvHsd females.^1^ The colony was expanded using heterozygous matings, and F1-F3 generations were used for experimental procedures. Wild type (*wt*) littermates were used as controls. Mice were genotyped for the *Cstb^tm1Rm^* mutation using genomic DNA from ear punch biopsies and confirmed with tail tissue DNA following euthanasia. The research protocols were approved by the Animal Ethics Committee of the State Provincial Office of Southern Finland (decisions ESAVI/10765/04.10.07/2015, ESAVI/10414/04.10.07/2017, ESAVI/13427/2018, and ESAVI/471/2019).

Mice were housed 2–5 animals per cage in non-reversed 12:12 h light/dark cycle with a 25 min dim period between the light and dark hours. Mice had *ad libitum* access to food and water in individually ventilated cages with air circulation 60–75 times per hour. Temperature in housing rooms was +23 ± 2°C and humidity 55 ± 15%. Experiments were performed at Orion Pharma (Turku, Finland).

**Figure 1:**
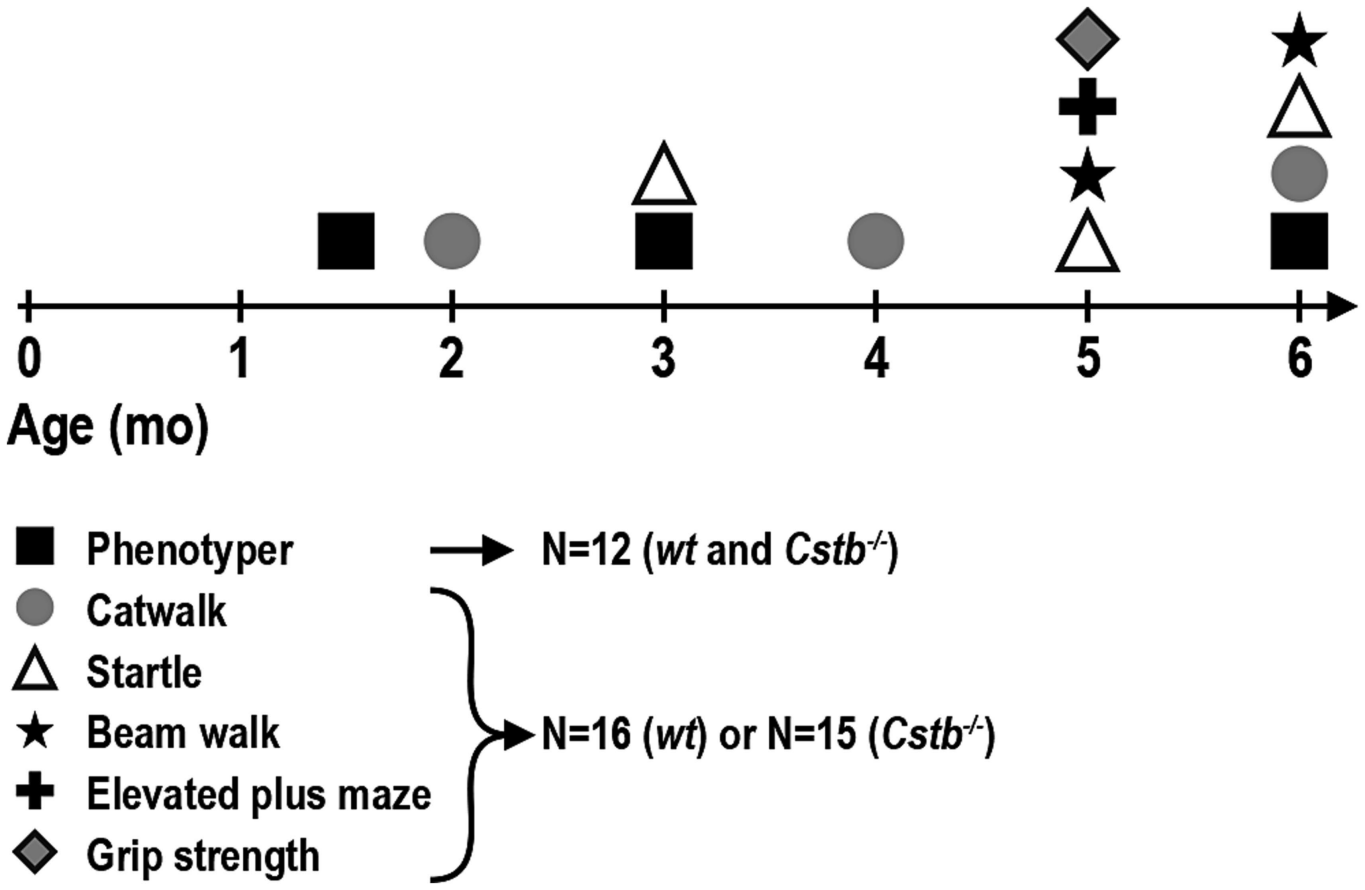
Timeline of the behavioral tests. Each animal completed all behavioral tests, except for Phenotyper that was performed only for a subset of animals: Phenotyper—myoclonus detection; CatWalk—walking pattern (coordination), speed of walk; Startle—startle response, prepulse inhibition; Beam walk—coordination; Elevated plus maze—hyperactivity, speed of walk; Grip strength—strength.

All behavioral tests were performed for the same cohort of mice at 6–27 weeks of age: *Cstb^−/−^* mice *N* = 15 (four females, 11 males) and wild type mice *N* = 16 (seven females, nine males), except for myoclonus detection, which was performed for a subset of these animals: *Cstb^−/−^* mice *N* = 12 (four females, eight males) and wild type mice *N* = 12 (six females, six males). Behavioral tests were conducted during the light phase of the circadian cycle between 8 a.m. and 3 p.m. Mice were habituated in their home cages in the testing room for a minimum of 30 min before starting the testing. Experimental design of the study depicting timepoints for different tests is outlined in Figure 1.

### 2.2 Myoclonus detection

Myoclonus detection was performed in Noldus PhenoTyper cages and analyzed with EthoVision XT 15 (Noldus) software. Up to 12 PhenoTyper cages were connected to one computer to enable simultaneous recording of 12 mice. The PhenoTyper 3000 cages have a floor area of 30 cm × 30 cm. Opaque walls were used for optimal video recording. The top units of the PhenoTyper cages are equipped with infra-red sensitive cameras that record the movement of the animals from above. For the best contrast, white floors with light colored bedding were used for brown mice. Cages always had bedding material (aspen chips). Cotton sticks or other nest making materials were not used as they interfered with the visibility of the mice during the recording. Mice had access to water and food throughout the measurements.

The recording of myoclonus lasted 4 h and was started at 10 a.m. After habituation in the testing room, mice were placed individually in the PhenoTyper cages, and the recording was set in the Trail control settings to start when the center-point of the animals was in the arena for more than 3 s. Mice were not disturbed during the recording and the female experimenter left the room for the duration of the recording. Mice were returned to their home cages after the recording.

The first hour of the recording was not used for myoclonus assessment as the mice were exploring the cage and moving more actively. Myoclonus in the *Cstb^−/−^* mouse are predominantly present and more reliably detected when the animals are sleeping (Pennacchio et al., 1998; Houseweart et al., 2003). Therefore, only the three last hours of the recording, when the animals were already habituated to the cage and mainly sleeping, were used for quantification.

The following setting were selected in EthoVision XT 15 software for the analysis profile:

Velocity: Outlier filter Averaging interval 1 sample, Body Points Center-point.
Multi Condition 1 conditions: Movement, Moving of the Center-point with threshold at 3.20 and 2.25 cm/s is true; Velocity, Velocity for the Center-point, averaged over 100 samples is ≤0.45 cm/s; Body elongation, Body elongation is ≤75.00%; Body angle, Body angle is ≤20.00 deg. All conditions are true.
Multi Condition 2 conditions: Acceleration, Acceleration of the Center-point is ≤180.00 cm/s^2^; Movement, Moving of Center-point, Nose-point and Tail-base, with thresholds at 2.00 and 1.75 cm/s is true. All conditions are true.
Mobility state: Outlier filter averaging interval 15 samples, Threshold Highly mobile above 60.00% and Immobile below 5.00%.

At the Integrated Visualization, the following parameters were used for the myoclonus detection: Velocity, Multi condition 1, Multi condition 2, and Mobility state. Time span was set to 30 s and the velocity scale was set to approx. 0–60 cm/s to visualize in the graph the mobility bursts caused by the myoclonus.

Positive myoclonus were seen as a short (1 s) activity burst (velocity ~ 20–60 cm/s) that were most often preceded by long immobile state and could be followed by slow movement while the animal was recovering from the event. In the Integrated visualization graphic presentation, myoclonus was identified by simultaneous break in the immobile state, short burst in velocity and positive detection in multi condition 1 and/or multi condition 2 (Supplementary Figure S1). Of note, not all events that fulfill the beforementioned criteria were myoclonus. Therefore, events that fulfilled the above-mentioned criteria in the Integrated visualization graphic presentation, were visually confirmed from the video recording. Events, where mice were clearly walking or moving voluntarily in the cage, were rejected and only the events that could be considered as involuntary myoclonic jerks were counted as true events. Examples of events classified as myoclonus can be seen in Supplementary Video S1. Software-assisted pre-screening of the possible positive events expedited the identification of the myoclonus as it eliminated the need to visually check over the full lengths of the video recordings.

The extent of the myoclonic event was relative to the size of the velocity burst in the graphic visualization. The parameter used for the analysis was total number of myoclonic events during the observation period.

### 2.3 Startle response detection

Startle response and prepulse inhibition (PPI) tests were performed using Startle Response (TSE Systems) operating on Startle Response/PPI Version 03.00 software.

Prepulse inhibition experiments were performed with following parameters: Background noise of 65 dB white noise was used throughout the experiment. Measurements consisted of 300 s habituation, 100 s baseline, five times 100 dB startle, and 10 blocks of three prepulse inhibition (PPI) trials and 100 dB startle alone presented in pseudorandom order with 10–30 s intertrial interval. Startle alone was 40 ms of 100 dB and 100 ms recording from the beginning of the noise. PPI trials consisted of 20 ms noise prepulse, 100 ms interstimulus interval, 40 ms 100 dB startle, and 100 ms recording from the beginning of startle. Used prepulse intensities were 69, 73, and 77 dB.

### 2.4 Elevated plus maze

Elevated plus maze consisted of four arms (two opposite open and enclosed arms) that were arranged to form a plus shape [dimensions: length (from the end of one arm to the end of another arm) 65 cm × 65 cm; width of the arm: 5 cm; height of the walls of the closed arms: 13 cm; apparatus was elevated 53 cm above the floor]. The bottom of the arms was gray, to increase the visibility of the brown colored mouse from the background and to avoid losing the signal of the camera during the observation. The recordings of the behavior were made with EthoVision XT 15 (Noldus) recording system with an attached infrared camera.

Each mouse was recorded for 5 min. Mouse was placed in the intersection of the four arms, the nose toward the same open arm each time. The mouse’s behavior was followed from a separate room. If the mouse fell off from the apparatus, it was placed back to the intersection, the nose toward the same open arm as in the start.

### 2.5 CatWalk

Walking pattern was assessed using CatWalk XT 10.6 automated gait analysis system (Noldus). In the test, mice traversed 120 cm long glass floor corridor. The footprints from the illuminated glass plate were recorded with a high-speed camera and data analyzed with CatWalk XT software.

### 2.6 Beam walk

The 1 m long beam was made of metal. The beam was elevated 50 cm above the bench by metal poles. The distance to travel in the test was 80 cm. The home cage was used as the goal box.

Before the first measurement at 5 months of age, mice were trained for 3 days. On the first training day, a beam with diameter of 10 mm was used. Each mouse was trained three times with a minimum of 30 s break between the trials. On the second and third trial day, training was performed first as on the first day using the 10 mm beam and then followed by training on an 8 mm beam. Before the second experimental time point (6 months), mice were trained only on 1 day and the training was repeated twice on both 10 and 8 mm beams.

The measurements on the experiment day were performed on the day following the training session. The mouse was placed at the start position of the 8 mm beam. The time to travel from the start to the end was measured. The measurement was repeated three times with a 30 s break. If the mouse failed to reach the end of the beam (i.e., fell off from the beam or turned upside down), the distance traveled was measured (10 cm accuracy). The cut off time for the measurements was 30 s. An average of three measurements was used for statistical analysis.

### 2.7 Grip strength

Grip strength was measured using grip strength meter (model 47105-001, Ugo Basile, Italy). The front paws of the mouse were placed on the metal bar and the mouse was gently pulled horizontally backwards from the base of the tail. The pull force on the mouse’s tail was increased until the mouse released the bar. An average of five trials was used for statistical analysis.

### 2.8 Statistical analyses

Statistical analyses were performed with GraphPad Prism (version 9.1.0, GraphPad Software, San Diego, CA). All data are presented as mean ± standard deviation (SD). Data were analyzed using two-way ANOVA followed by Šídák’s multiple comparisons test to determine difference between groups. Two-way repeated measures ANOVA was used where applicable. Difference between groups was analyzed by unpaired *t*-test for grip strength and elevated plus maze measurements. Statistical significance was set at a value of *p* < 0.05 (*), additional presentation of significance with asterisks are value of *p* < 0.01 (^**^), value of *p* < 0.001 (^***^), and value of *p* < 0.0001 (^****^).

## 3 Results

### 3.1 *Cstb*^−/−^ mice have progressive myoclonus

**Figure 2:**
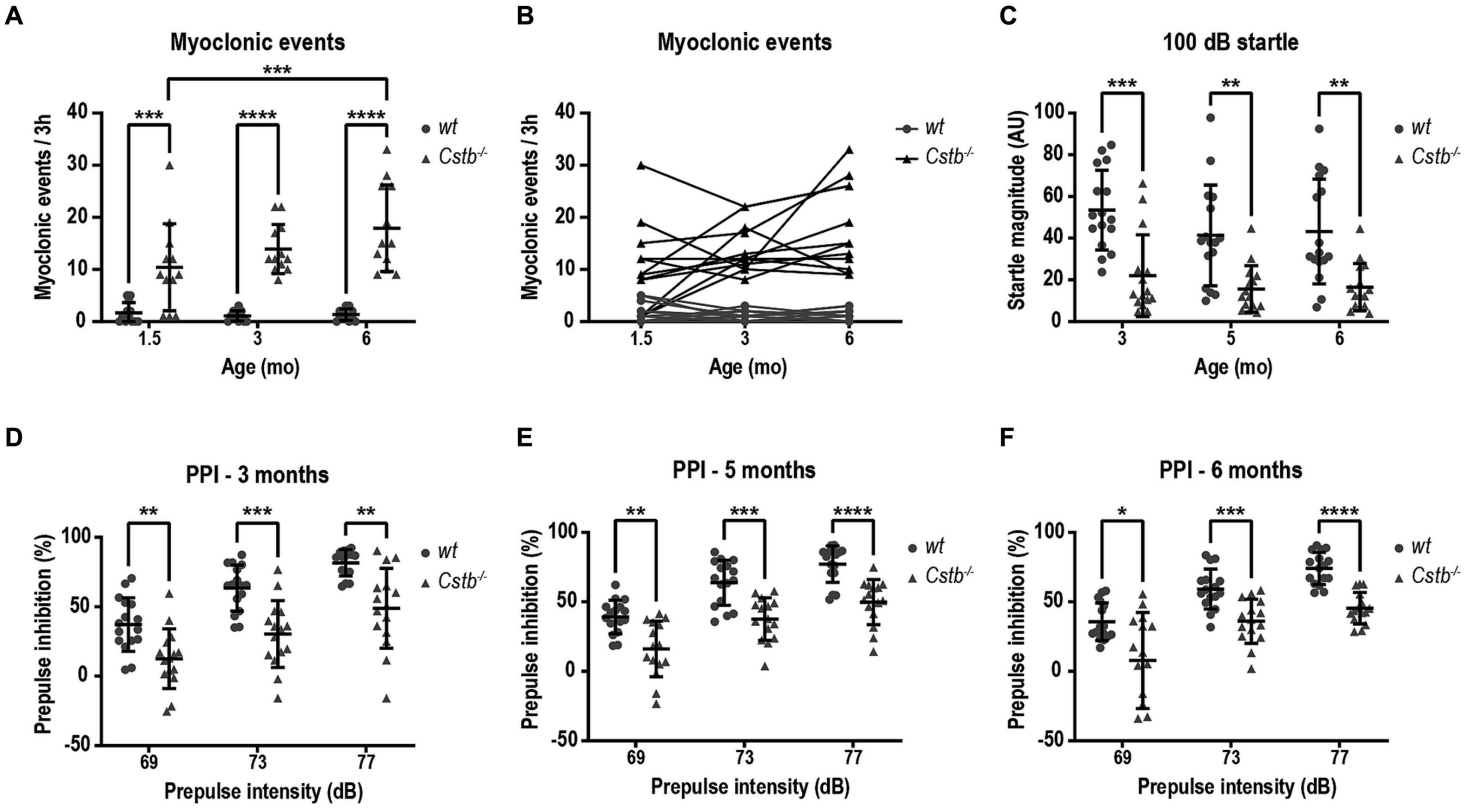
*Cstb^−/−^* mice show progressive myoclonus and altered startle response. **(A)** Myoclonic events were detected in PhenoTyper cages for a period of 3 h at 1.5, 3, and 6 months of age. **(B)** Progression of myoclonic events from 1.5 to 6 months of age in individual animals (see Supplementary Figure 2 for separate graphs of *Cstb^−/−^* mice). **(C)** *Cstb^−/−^* mice show reduced startle response to 100 dB noise at 3, 5, and 6 months of age compared to the wild type (*wt*) mice. **(D–F)** Prepulse inhibition (PPI) at 69, 73, and 77 dB prepulse intensities is decreased in *Cstb^−/−^* mice at the age of 3 **(D)**, 5 **(E)**, and 6 **(F)** months. Data are presented as mean ± SD. ^*^p < 0.05, ^**^p < 0.01, ^***^p < 0.001, and ^****^p < 0.0001, *N* = 12 for **(A,B)**, *N* = 15–16 for **(C–F)**.

To assess the presence and frequency of myoclonus in non-disturbed environment, mice were monitored by video recording in PhenoTyper cages. The number of myoclonic events was assessed for duration of 3 h during the light cycle of the circadian rhythm, i.e., during the resting phase of mice. Most often, myoclonic jerks were isolated events during sleep. In the video recording, the myoclonus could be seen as a jump in the air or, more modestly, as a nod to the side or jerk forward (Supplementary Video S1). Usually, after the event, the mouse was moving around for a short period of time until it returned to sleep. Occasionally, there were two consecutive myoclonic events separated by a 1–20 s interval and often one of these was notably larger in magnitude. Myoclonic events were detected in *Cstb^−/−^* mice already shortly after weaning at the age of 1.5 months (Figure 2A). Compared to wild type, *Cstb^−/−^* mice had significantly more myoclonic events at all measured time-points [*F*(1, 44) = 70.71, 1.5 months *p* = 0.0004, 3 months *p* < 0.0001, and 6 months *p* < 0.0001] and the number of myoclonic events increased in *Cstb^−/−^* mice from age of 1.5 to 6 months [*F*(2, 44) = 4.373, *p* = 0.0004, Figure 2A]. The number of myoclonic events in individual *Cstb^−/−^* mice remained similar or increased in most animals over time (Figure 2B; Supplementary Figure S2). Some wild type mice had myoclonic-type twitches, but these events were less frequent than events in *Cstb^−/−^* mice.

### 3.2 *Cstb*^−/−^ mice show defects in startle response

Clinical observations imply that EPM1 patients may have altered startle response and furthermore, patients can respond strongly to auditory and visual stimuli as these can evoke myoclonus (Lehesjoki and Kälviäinen, 2020). To investigate potential defects in sensorimotor gating mechanisms, the mice were assessed for prepulse inhibition of an acoustic startle response following 69, 73, and 77 dB prepulses. Compared to wild type mice, the magnitude of the startle response was diminished in *Cstb^−/−^* mice from the first measurement at the age of 3 months to the last observation at the age of 6 months [*F*(1, 29) = 23.13, 3 months *p* = 0.0003, 5 months *p* = 0.0027, and 6 months *p* = 0.0027, Figure 2C]. The reduced startle response was accompanied by diminished prepulse inhibition at all three different prepulse intensities and at all measured ages [at 3 months *F*(1, 29) = 21.54, 69 dB *p* = 0.0068, 73 dB *p* = 0.0005, and 77 dB *p* = 0.0018, at 5 months *F*(1, 29) = 27.50, 69 dB *p* = 0.0024, 73 dB *p* = 0.0002, and 77 dB *p* < 0.0001, and at 6 months *F*(1, 29) = 26.53, 69 dB *p* = 0.0267, 73 dB *p* = 0.0007, and 77 dB *p* < 0.0001, Figures 2D–F].

### 3.3 *Cstb*^−/−^ mice have motor coordination problems that do not resemble ataxia

**Figure 3:**
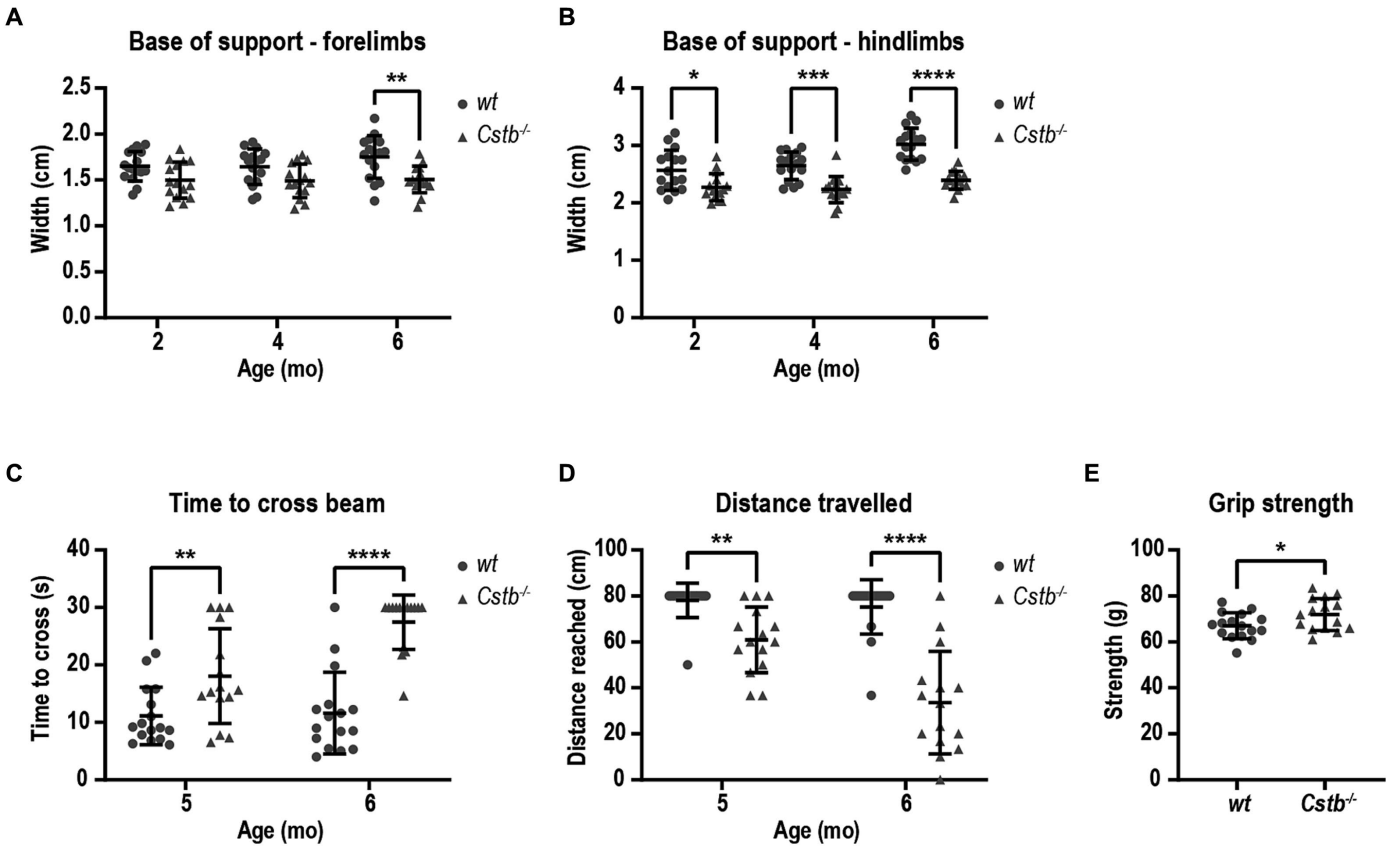
*Cstb^−/−^* mice have motor coordination problems that do not resemble ataxia. Base of support is equal or narrower in *Cstb^−/−^* mice in comparison to wild type (*wt*) mice both for forelimbs **(A)** and hindlimbs **(B)** in contrast to widening gait usually seen in ataxic phenotype. **(C)** *Cstb^−/−^* mice need longer time to cross a 80 cm beam of 8 mm in diameter at the age of 5 and 6 months, **(D)** and fail more often to travel the whole 80 cm distance on the beam. **(E)** The grip strength of front paws is not reduced in 5-month-old *Cstb^−/−^* mice compared to wild type mice. Data are presented as mean ± SD. ^*^p < 0.05, ^**^p < 0.01, ^***^p < 0.001, and ^****^p < 0.0001, *N* = 15–16.

As both EPM1 patients and *Cstb^−/−^* mice have been reported to show ataxic gait, we wanted to evaluate the motor deficit in *Cstb^−/−^* mice in more detail with CatWalk system which is a sensitive and quantitative method for this purpose. The walking pattern was assessed using the automatic gait analysis system CatWalk at 2, 4, and 6 months of age. Ataxic gait can be characterized by wider distance between adjacent paws which occurs as a compensatory mechanism to maintain balance (Kyriakou et al., 2016). The base of support parameter in CatWalk that measures the distance between the center points of the two fore or hind paws was found to be similar or smaller in both fore- and hindlimbs of *Cstb^−/−^* mice in comparison to wild type mice at all measured time points [forelimbs *F*(1, 29) = 12.02, 2 months *p* = 0.0826, 4 months *p* = 0.0875, and 6 months *p* = 0.0048, hindlimbs *F*(1, 29) = 35.35, 2 months *p* = 0.0292, 4 months *p* = 0.0001, and 6 months *p* < 0.0001; Figures 3A,B].

To further evaluate the motor performance and balance, mice were assessed using beam balance test at 5 and 6 months of age. The beam walk test detects sensorimotor and cortical deficits and therefore offers an additional insight into the characteristics of the motor problems. *Cstb^−/−^* mice needed longer time to cross the beam and more frequently fell from the beam without being able to travel the whole distance of 80 cm [time to cross the beam *F*(1, 29) = 40.35, 5 months *p* = 0.0080 and 6 months *p* < 0.0001, distance *F*(1, 29) = 53.11, 5 months *p* = 0.0041, 6 months *p* < 0.0001; Figures 3C,D]. Worsening of the motor performance was observed in as short time frame as 1 month. By visual inspection, *Cstb^−/−^* mice had clear problems in motor performance as they made frequent slips from the beam with hind paws, and they wrapped the tail around the beam to gain more balance unlike control mice (Supplementary Video S2). The problems in motor performance were not related to reduced grip strength of the forelimbs (*t* = 2.159, df = 29, *p* = 0.0393; Figure 3E).

### 3.4 *Cstb*^−/−^ mice are hyperactive and lack inhibition

**Figure 4:**
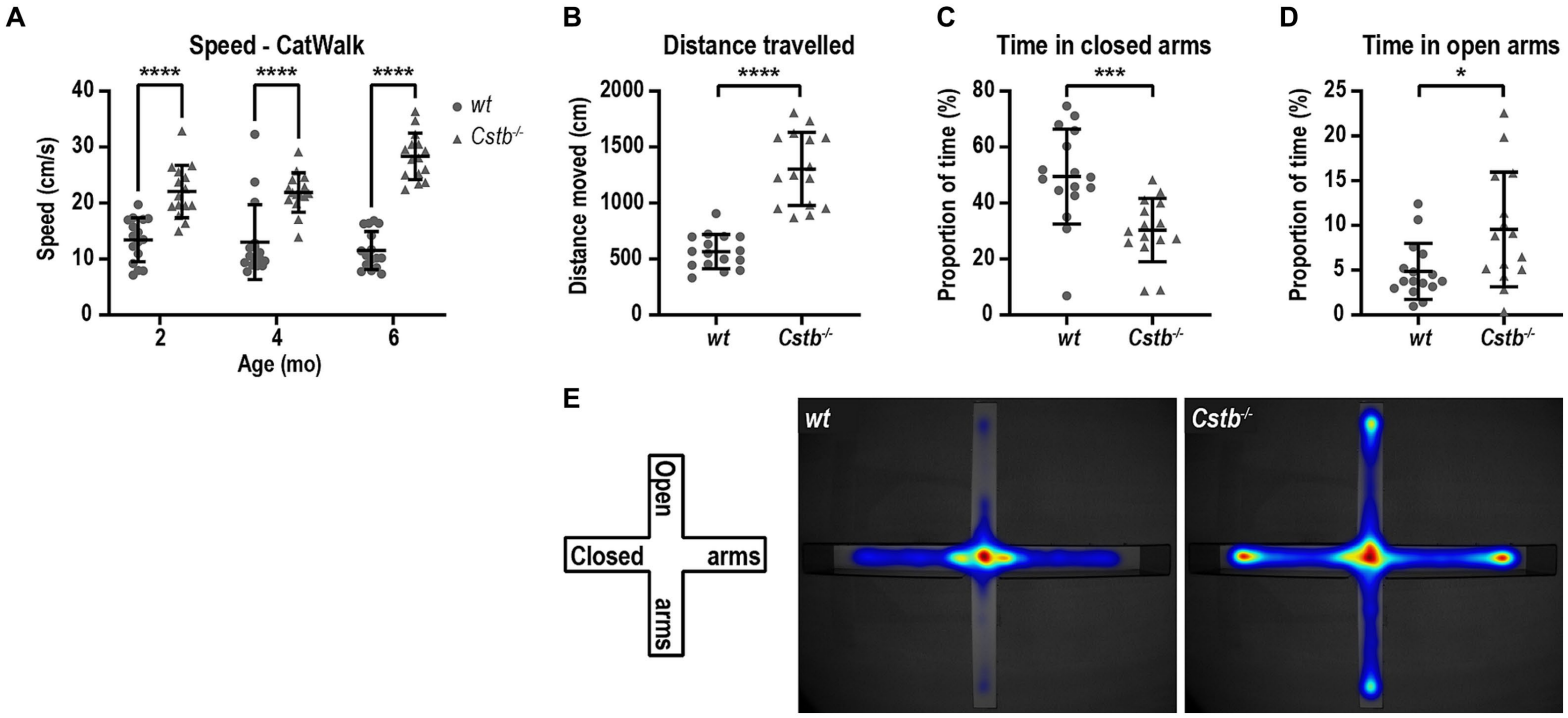
*Cstb^−/−^* mice show a hyperactive phenotype. **(A)** The 2-, 4-, and 6-month-old *Cstb^−/−^* mice move faster in CatWalk test compared to the wild type (*wt*) mice. **(B–D)** In elevated plus maze test, 5-month-old *Cstb^−/−^* mice travel longer distance during the 5 min observation period **(B)** and spend less time in the closed arms **(C)** and more time in the open arms **(D)** than wild type mice. **(E)** Pooled heat map of activity for wild type and *Cstb^−/−^* mice in the elevated plus maze. Data are presented as mean ± SD. ^*^p < 0.05, ^***^p < 0.001, and ^****^p < 0.0001, *N* = 15–16.

CatWalk analysis revealed that the *Cstb^−/−^* mice had greater average speed than wild type littermates at all measured ages [*F*(1, 29) = 141.0, 2 months *p* < 0.0001, 4 months *p* < 0.0001, and 6 months *p* < 0.0001; Figure 4A] which prompted us to investigate the activity of the mice in more detail. Elevated plus maze was chosen to evaluate the activity of the mice as it assesses not only the general activity but also risk assessment and inhibition. At the age of 5 months, *Cstb^−/−^* mice were hyperactive and traveled more than twice the distance that wild type mice did during the 5 min observation period (*t* = 8.145, df = 29, *p* < 0.0001; Figure 4B). Additionally, *Cstb^−/−^* mice exhibited reduction in risk assessment as they spent less time in closed arms and more time in open arms than wild type mice (closed arms *t* = 3.675, df = 29, *p* = 0.0010, open arms *t* = 2.610, df = 29, *p* = 0.0142; Figures 4C–E).

The behavioral characteristics of the *Cstb^−/−^* mice that have been identified here and in previous publications are summarized in Table 1 and compared to symptoms of the EPM1 patients.

**Table 1:** Comparison of the symptoms in EPM1 patients and in *Cstb^−/−^* mice.

EPM1 patients	*Cstb^−/−^* mice
Myoclonic jerks during wakefulness and sleep from 6–15 years of age^1^	Myoclonic events from 1.5 month of age during sleep
Motor coordination impairment^1^	Motor coordination impairment
Emotional instability, impulsivity, depression, and anxiety^2^	Hyperactivity, agitation, and lack of inhibition
Tonic–clonic seizures^1^	Tonic–clonic seizures absent
Affected sensorimotor gating: decreased startle response, repetition suppression reduced^3,4^	Affected sensimotor gating: reduced prepulse inhibition and startle response

^1^Lehesjoki and Kälviäinen (2020), ^2^Kälviäinen (2015), ^3^Kiziltan et al. (2017), and ^4^Julkunen et al. (2018).

## 4 Discussion

The understanding of symptoms and disease progression in EPM1 patients has increased over the past two decennia because of active clinical research in genetically verified patient cohorts. The *Cstb^−/−^* mouse model of EPM1, introduced 25 years ago (Pennacchio et al., 1998), has been the target of active research regarding the underlying histological, cellular, and molecular pathology associated with CSTB-deficiency. However, reports on the behavioral phenotype of *Cstb^−/−^* mice are scarce and have been mainly done in mixed genetic background. To define the occurrence of myoclonus and behavioral deficits during disease progression up to 6 months of age in *Cstb^−/−^* mice, we here report a systematic analysis of mice in pure isogenic background.

The onset of symptoms in patients is around 6–15 years of age starting with progressively worsening myoclonus and/or tonic–clonic seizures followed by other motor coordination problems (Lehesjoki and Kälviäinen, 2020). This onset age corresponds to approximately the age of 20–40 days in mice (Bell, 2018). Thus, we began to study the *Cstb^−/−^* mice shortly after weaning to correlate the findings to pathophysiology in humans. Already in the first measurement at age of 1.5 months, nearly all *Cstb^−/−^* mice had repeated myoclonic events during the three-hour observation period. Consistent with the progressive nature of myoclonus in EPM1 patients and previous reports in mice (Pennacchio et al., 1998), the number of myoclonic events increased and became evident in all *Cstb^−/−^* mice as they aged. Some wild type mice had myoclonus-like events that presumably are physiological reflexes occurring while falling asleep. These twitches seemed to be more frequent in younger animals and were always modest in magnitude.

As the myoclonic events in the *Cstb^−/−^* mice in isogenic Sv129 background have previously been reported to occur only during sleep (Pennacchio et al., 1998), we limited the analysis to the period when the mice were mainly sleeping. We cannot, however, exclude the possibility of occurrence of the myoclonus during wakefulness and activity, as has been described in mice older than 8 months in mixed background (Houseweart et al., 2003). Detecting small twitches when the mice are moving is considerably more difficult than during inactivity. The detection parameters in the EthoVision software were specifically designed to distinguish the sudden bursts of activity during the period of general inactivity. The recording was not coupled to electrocorticographic recording and relied solely on the activity detection parameters of the EthoVision software and visual confirmation of the myoclonic events. Despite the limited resolution of the video recordings and the absence of the electrocorticographic recordings, the method was still effective on picking up the events from the video and we can recommend this method to be used in laboratories that do not have capability to perform intracranial measurements of cortical activity in mice. This novel method allows systematic quantitative assessment of myoclonus in contrast to previously published descriptive reports. The possibility to quantitatively measure the myoclonus enables the evaluation of the progression of the symptoms in the mice. Moreover, method allows assessment of the effect of therapeutic interventions and development of new treatments for myoclonus.

At all studied ages, *Cstb^−/−^* mice had significantly lower startle response compared to wild type mice. Startle response has not been extensively studied in EPM1 patients. However, a study in a group of progressive myoclonus epilepsy patients of different etiologies, including also EPM1 patients, reported decreased response to auditory startle in the patient group (Kiziltan et al., 2017). This observation suggests that progressive myoclonus epilepsy patients have a hypoactive startle response to somatosensory inputs concordant with findings in *Cstb^−/−^* mice. Our data further strengthen the previously implied mechanism of subcortical dysfunction in EPM1 (Julkunen et al., 2018).

Further analysis of the somatosensory responses indicated that prepulse inhibition was markedly reduced in *Cstb^−/−^* mice. In EPM1 patients, neural processing of external stimuli has been explored through short-term adaptation of the motor cortex to transcranial magnetic stimulation via repetition suppression phenomenon (Julkunen et al., 2018). Repetition suppression was mild or missing in EPM1 patients and abnormal response correlated with the myoclonus severity. Interestingly, repetition suppression was more severely altered in adults than in adolescent patients indicating progressive nature of the symptoms. Findings both in *Cstb^−/−^* mice and EPM1 patients point to insufficient adaptive reactiveness to stimuli and to somatosensory gating defects. The underlying cause for these defects may arise from impaired function of the thalamocortical system and alterations in GABAergic and dopaminergic systems (Silvennoinen et al., 2023).

Ataxic gait has been reported in EPM1 patients (Lehesjoki and Kälviäinen, 2020). For the purpose of detailed evaluation of possible ataxia in *Cstb^−/−^* mice, CatWalk setup was utilized as it can measure several gait parameters that can differentiate motor problems. In our study, the parameter depicting the ataxic gait, base of support, was not altered in *Cstb^−/−^* mice in the direction that would indicate ataxia. In ataxia, the distance between the adjacent paws becomes wider but in *Cstb^−/−^* mice we observed the opposite change. This indicates that the gait of the *Cstb^−/−^* mice is altered, but that this is likely to be caused by other motor coordination problems than ataxia. To analyze the motor performance in more detail, mice were challenged with a beam walk test where they had to walk on a narrow metal beam. The test is sensitive to detect problems in motor coordination and is used to detect sensorimotor and cortical deficits (Piot-Grosjean et al., 2001; Stanley et al., 2005). Furthermore, contrary to rotarod, beam walk is not susceptible for training effect. The problems with balance and coordination were evident in *Cstb^−/−^* mice. When mice attempted to traverse the beam, there were constant slips of the hindlimbs, and mice wrapped their tail around the beam to gain more support. The problems were reflected in the decreased speed while traversing the beam and in the inability to travel the whole distance without falling. As the grip strength of the *Cstb^−/−^* mice was not reduced, the problems can be presumed to be originating from impaired motor coordination. Maintained grip strength is in line with the clinical picture of EPM1 which does not include muscle involvement (Lehesjoki and Kälviäinen, 2020).

An unexpected finding in the CatWalk analysis was that *Cstb^−/−^* mice walked markedly faster than wild type mice already at the age of 2 months and this difference was persistent and even became more prominent until the age of 6 months. Thus, despite having motor problems, *Cstb^−/−^* mice were capable of walking fast on even surface. The finding was consolidated by the elevated plus maze test where *Cstb^−/−^* mice traveled more than twice the distance that the wild type mice did during the 5 min observation period. Moreover, *Cstb^−/−^* mice spent more time in the open arms whereas wild type mice avoided open elevated surfaces and preferred closed environment. Together, these observations suggest that *Cstb^−/−^* mice are hyperactive and lack inhibition in their behavior. Interestingly, EPM1 patients have various psychosocial challenges and psychiatric comorbidities including emotional instability, impulsivity, depression, and anxiety (Kälviäinen, 2015). It is thus tempting to speculate that these could reflect a lack of inhibition phenotype identified in *Cstb^−/−^* mice.

In previous reports (Pennacchio et al., 1998; Houseweart et al., 2003; Kaur et al., 2010), motor symptoms of *Cstb^−/−^* mice were studied using a limited set of experimental setups. These studies were conducted both on mixed and pure 129Sv genetic backgrounds. *Cstb^−/−^* mice were deduced to have ataxia based on the assessment on rotarod performance on a still or slowly rotating rod. *Cstb^−/−^* mice spent shorter time on the rod than wild type mice, which was interpreted as ataxic symptom. Our findings from more refined tests on *Cstb^−/−^* mice in pure isogenic 129S2/SvHsd background up to 6 months of age indicate that these mice have motor deficits but that these are not due to ataxia. Absence of an ataxic phenotype is somewhat unexpected given the striking cerebellar atrophy reported in the *Cstb^−/−^* mice (Tegelberg et al., 2012; Manninen et al., 2013). *Cstb^−/−^* mice were found to be hyperactive, which was evident in several behavioral tests in our study. Therefore, the poor performance of *Cstb^−/−^* mice on rotarod reported by Pennacchio and co-workers could be the consequence of the mice being too agitated and impulsive to stay on a very slowly moving rod and they rather escape from the rod due to hyperactivity than fall from it due to ataxia.

The key phenotype in EPM1 is drug-refractory myoclonus, which, contrary to *Cstb^−/−^* mice, appears in addition to sleep also during wakefulness as stimulus-sensitive myoclonic jerks with action-induced myoclonus being particularly characteristic (Rissanen et al., 2021). Ataxic gait has been also reported in EPM1-patients although it is very challenging to differentiate the effects of action myoclonus from ataxia in motor examination in EPM1 and the atactic features improve or cease if the myoclonus is better controlled with treatment. Difficulty in starting to walk and insecure movement due to possibility to fall because of negative myoclonus may appear as ataxic gait. Indeed, careful evaluation of the motoric performance in EPM1 patients supports the idea that also in EPM1 patients the gait problems are caused by myoclonus and other motor coordination problems but not ataxia.

As EPM1 patients suffer cumulatively from several different symptoms during disease progression, the *Cstb^−/−^* mouse model enables the testing and development of new treatments for specific symptoms. In particular, the severe myoclonus remains a treatment challenge in most patients. The newly identified phenotypic features combined with the methods described in this study provide novel tools for drug development allowing quantitative assessment when testing the efficacy of drugs for different symptoms. The *Cstb^−/−^* model enables studies to understand the neuropathological processes in parallel with the occurrence of the symptoms and may even help the development of disease modifying treatments.

Taken together, our detailed behavioral phenotyping of the *Cstb^−/−^* mice reveals new aspects of this mouse model. Similarly to human EPM1 patients, the most prominent symptom in the *Cstb^−/−^* mice is adolescent onset progressive myoclonus that interferes with motor coordination. In addition, adolescent *Cstb^−/−^* mice have hyperactivity and lack of inhibition similarly as is currently increasingly diagnosed in adolescent EPM1 patients showing impulsivity, anxiety and emotional instability. The nature of the phenotype in the *Cstb^−/−^* mice seems to be more complex than initially suggested and more resembling the human phenotype than initially described. Furthermore, hyperactivity and lack of inhibition are novel findings from the mouse model that can lead to re-evaluate the behavior of the patients as detailed evaluation of their behavioral and neuropsychiatric symptomatology has been ignored. A detailed phenotyping of the mouse model that has been created 25 years ago can still provide novel insights and understanding on the complex genetic disorder and offer valuable new details that are valid for understanding the clinical features of the disease.

## Data availability statement

The original contributions presented in the study are included in the article/Supplementary material, further inquiries can be directed to the corresponding author.

## Ethics statement

The animal study was approved by Animal Ethics Committee of the State Provincial Office of Southern Finland. The study was conducted in accordance with the local legislation and institutional requirements.

## Author contributions

EP: Conceptualization, Data curation, Formal analysis, Investigation, Methodology, Supervision, Visualization, Writing – original draft, Writing – review & editing. ST: Conceptualization, Data curation, Formal analysis, Investigation, Methodology, Resources, Visualization, Writing – original draft, Writing – review & editing, Supervision. HB: Conceptualization, Project administration, Supervision, Writing – review & editing. RK: Conceptualization, Data curation, Investigation, Writing – review & editing. A-EL: Conceptualization, Data curation, Investigation, Project administration, Resources, Supervision, Writing – original draft, Writing – review & editing, Funding acquisition. AH: Conceptualization, Data curation, Funding acquisition, Investigation, Methodology, Project administration, Resources, Supervision, Writing – review & editing.

## Footnotes

^1^ https://www.inotivco.com/model/129s2-svhsd
