# Supplementary material for: In depth behavioral phenotyping unravels complex motor disturbances in Cstb−/− mouse, a model for progressive myoclonus epilepsy type 1
Source: Front Behav Neurosci. 2023 Dec 21;17:1325051. doi: 10.3389/fnbeh.2023.1325051 (PMC10764494; doi:10.3389/fnbeh.2023.1325051)
Supplement: Supplementary file 3 [file Image_1.pdf]

Myoclonus

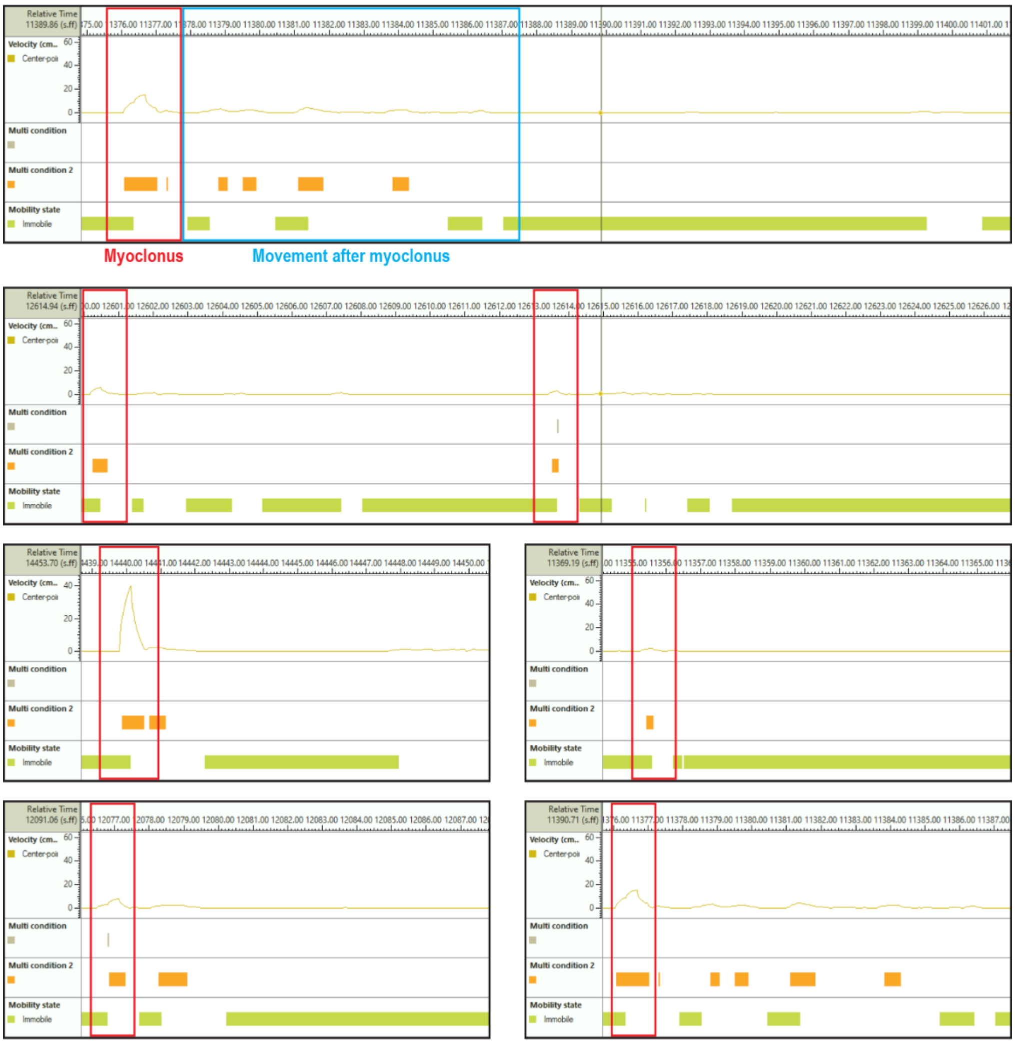

Normal movement

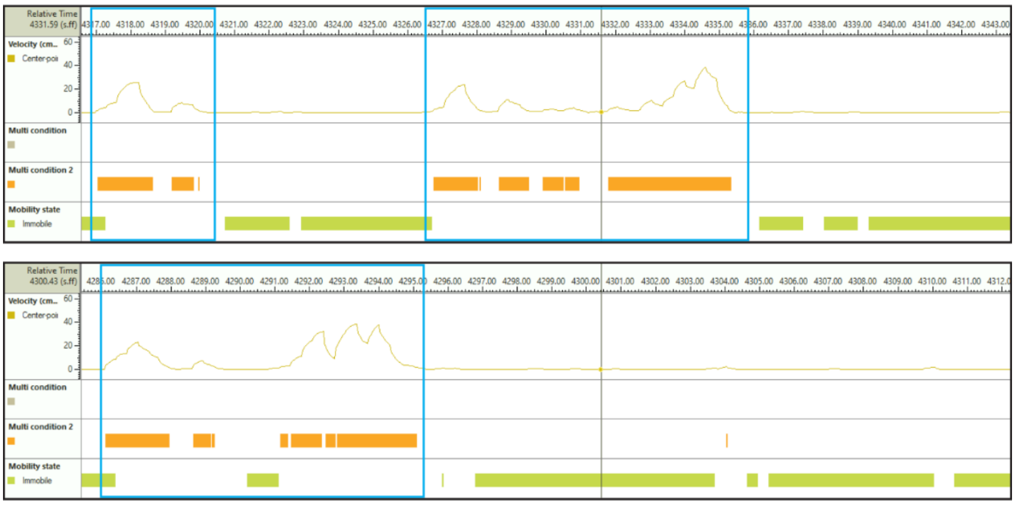

Supplementary Figure 1. Representative views of movement in EthoVision software, recorded from PhenoTyper cages. Yellow line represents the velocity of the animal's center point, grey and orange bars denote the times when conditions 1 and 2 are fulfilled, respectively, and green bars mark times of immobility. Myoclonus is marked with red squares and normal movement with blue squares.

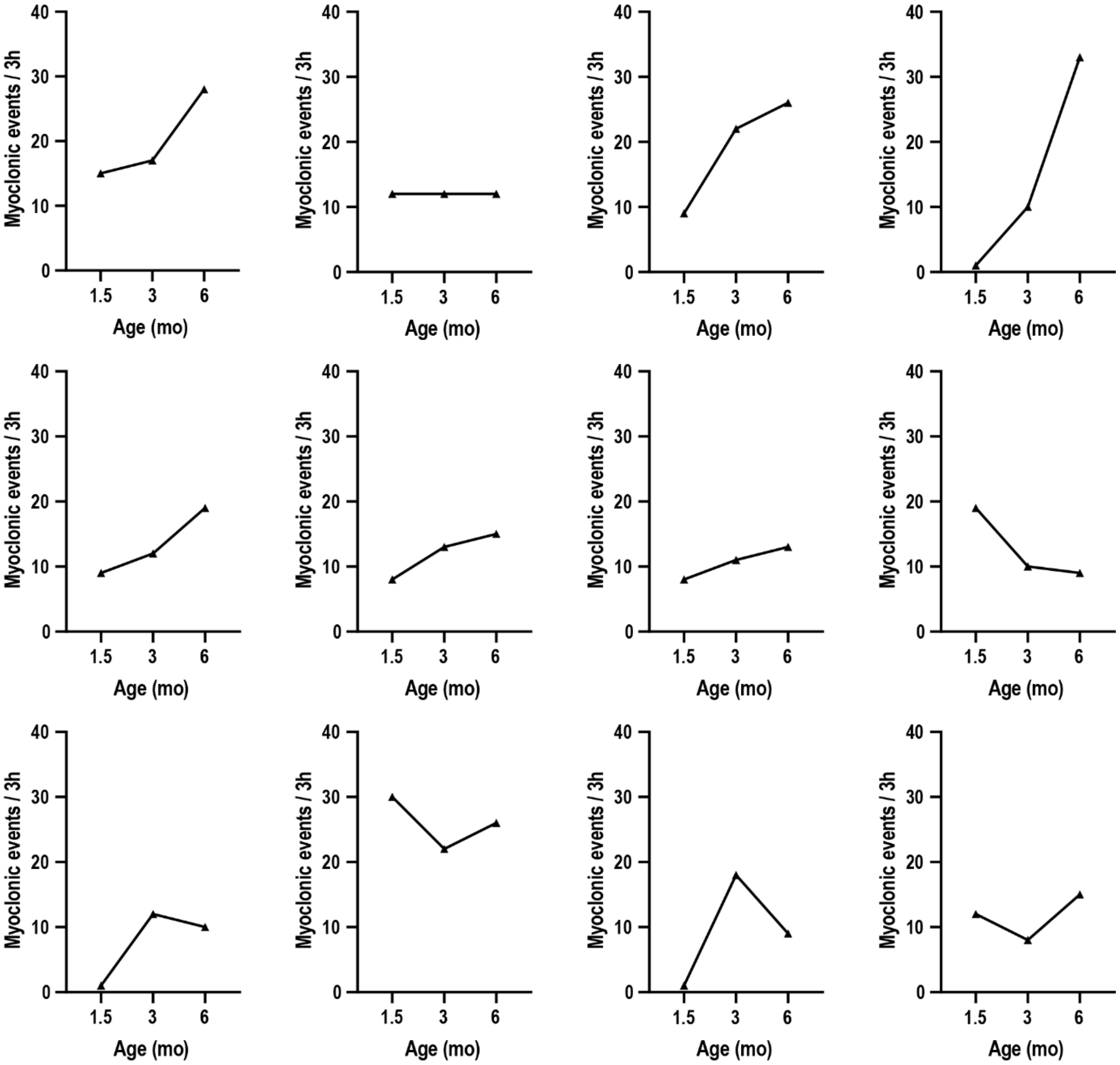

Supplementary Figure 2. Progression of myoclonic events from 1.5 to 6 months of age in individual *Cstb*$^{-/-}$ mice, detected in PhenoTyper cages for a period of 3 hours.

Supplementary Video 1. Representative videos of myoclonic events, recorded in PhenoTyper cages.

Supplementary Video 2. Representative beam walk performances of control and *Cstb*$^{-/-}$ mice (80 cm long, 8 mm diameter beam).
